# Elevated Interleukin-36α And CD4^+^IL-36α^+^T Cells Are Involved in the Pathogenesis of Graves' Disease

**DOI:** 10.3389/fendo.2018.00591

**Published:** 2018-10-09

**Authors:** Qiu-ming Yao, Ling Li, Zhen-Yu Song, Bin Wang, Qiu Qin, Xiao-fei An, Jin-an Zhang

**Affiliations:** ^1^Department of Endocrinology, Jinshan Hospital of Fudan University, Shanghai, China; ^2^Department of Urology, Jinshan Hospital of Fudan University, Shanghai, China; ^3^Department of Endocrinology, The Affiliated Hospital of Nanjing University of Chinese Medicine, Nanjing, China

**Keywords:** graves' disease, interleukin-36α, CD4^+^IL-36α^+^T cells, thyroid, cytokines

## Abstract

**Background:** IL-36α is involved in the pathogenesis of a variety of autoimmune diseases, but the relationship between IL-36α and Graves' disease (GD) has rarely investigated. In the present study, we aimed to explore the expression of IL-36α and elucidate the potential role of IL-36α in GD.

**Methods:** The expression of IL-36α mRNA in peripheral blood mononuclear cells (PBMCs) from 32 newly diagnosed GD patients, 15 refractory GD patients and 30 normal controls (NC) was examined using quantitative real-time polymerase chain reaction (qRT-PCR). The level of IL-36α in serum from 46 newly diagnosed GD patients, 10 refractory GD patients and 24 NC was measured using enzyme linked immunosorbent assay (ELISA). The percentage of CD4^+^IL-36α^+^T cells was detected by flow cytometry. PBMCs from newly diagnosed GD patients and NC group were cultured in the presence or absence of recombinant human IL-36α, and the expression levels of IFN-γ, TNF-α, IL-6, and IL-17A in culture supernatant were detected by cytokine array.

**Results:** The expression of IL-36α mRNA in newly diagnosed GD patients was significantly higher than that in NC group (*P* = 0.019). IL-36α mRNA expression was positively associated with thyrotropin receptor antibody (TRAb) (*P* = 0.004, *r* = 0.498) in newly diagnosed GD patients. The level of IL-36α in serum from newly diagnosed GD patients was significantly higher than that in refractory GD patients and NC group (*P* = 0.01; *P* = 0.007). The percentage of CD4^+^IL-36α^+^T cells in newly diagnosed GD patients was significantly higher than that in NC group (*P* = 0.030). In GD group, recombinant human IL-36α stimulation resulted in the increase of INF-γ, TNF-α, IL-6 and IL-17A (*P* = 0.015; *P* = 0.016; *P* = 0.039; *P* = 0.017).

**Conclusion:** IL-36α and CD4^+^IL-36α^+^T cells may be involved in the pathogenesis of GD by promoting the production of Th1, Th2, and Th17 cytokines.

## Introduction

Graves' disease (GD), also known as the diffuse toxic goiter, is a common subtype of autoimmune thyroid disease (AITD). As an organ-specific autoimmune disease, GD is characterized by hyperthyroidism and a large amount of thyrotropin receptor antibody (TRAb) in serum. GD is the most common cause of hyperthyroidism, and its annual incidence is approximately 20–50/100,000 (1). In addition to hyperthyroidism syndrome, GD may also accompany some clinical manifestations outside thyroid, mainly including ophthalmopathy, pretibial myxedema and clubbing. GD can be observed in any age, but more frequently encountered in women at the productive age. The risk ratio of GD between male and female is about 1: 6 (2). Previous studies from our and other research teams have documented that the interruption of classical Th1/Th2 cell balance, and newly defined Th17/Treg cell balance, as well as newly discovered lymphocytes such as Th22 cells and follicular helper T (Tfh) cells are involved in the development of GD (3–8), but so far, the specific pathogenesis of GD is still unclear.

IL-36α is a new member of the IL-1 family (9), also known as IL-36A or IL-1F6. IL-36α, IL-36β (IL-1F8) and IL-36γ (IL-1F9) are collectively referred to as IL-36 due to the same biological function (10). IL-36α can execute pro-inflammatory and immunomodulatory functions through binding its heterodimeric receptors consisting of IL-36R and IL-1 receptor accessory protein (IL-1R/AcP), and activating mitogen-activated protein kinase (MAPK) and transcription factor NF-kB signaling cascades (11). As a kind of pro-inflammatory factor, IL-36 is expressed in many tissues, with the most notably expression in skin, esophagus, tonsils, lungs, bowel, and brain; in addition, IL-36 can also be expressed in immune cells, including monocytes, macrophages and T cells (12). Because IL-36R expressed in human monocytes, IL-36 can stimulate human monocyte-derived dendritic cells (MDC) to produce a variety of pro-inflammatory cytokines, including IL-1, IL-6 IL-12, IL-12, and IL-18, and can also enhance the expression of CD83 and MHC-II on the cell surface, thus promoting MDC maturation; vice versa, under the stimulation of IL-36, MDC can promote T cell proliferation and IFN-γ production (13, 14). It is found that IL-36α and IL-36β can up-regulate the production of pro-inflammatory cytokines such as TNF-α and IL-17; in turn, TNF-α, IL-17 and IL-22 can also stimulate the expression of IL-36 (15). IL-36 may not only act solely on naive CD4^+^ T cells and promote its proliferation and IL-2 expression, but also may execute a synergistic effect on Th1 polarization when combined with IL-12 (16).

Previous studies have confirmed that IL-36α is involved in the pathogenesis of a variety of autoimmune diseases such as rheumatoid arthritis, Sjogren's syndrome, inflammatory bowel disease, and so on, but the relationship between IL-36α and GD has rarely been investigated. Therefore, in the present study, the possible role of IL-36α and underlying mechanism in the pathogenesis of GD were explored.

## Materials and methods

### Subjects

In the present study, all GD samples were collected from the Department of Endocrinology, Jinshan Hospital of Fudan University and used as the case group, the normal controls with matched sex and age of the case group were selected from the Physical Examination Center of the same hospital. All subjects included in this study signed informed consent. In this study, 134 GD patients and 73 normal controls (NC) were included. As shown in Supplementary Table 1, among them, 32 newly diagnosed GD patients (7 males and 25 females, 37.6 ± 13.7 years old), 15 refractory GD patients (3 males and 12 females, 33.6 ± 11.1 years old), and 30 normal controls (8 males and 22 females, 35.2 ± 12.3 years old) were collected for quantitative real-time polymerase chain reaction (qRT-PCR); 46 newly diagnosed GD patients (15 males and 31 females, 43.8 ± 12.4 years old), 10 refractory GD patients (5 males and 5 females, 41.3 ± 9.4 years old) and 24 normal controls (8 males and 16 females, 40.7 ± 9.8 years old) were collected for ELISA; 19 newly diagnosed GD patients (3 males and 16 females, 41.0 ± 9.2 years old) and 10 normal controls (2 males and 8 females, 38.2 ± 6.0 years old) were collected for flow cytometry. Twelve patients with newly diagnosed GD (4 males and 8 females, 36.7 ± 11.6 years old) and 9 normal controls (2 males and 7 females, 35.3 ± 9.0 years old) were collected for cell stimulation. Newly diagnosed GD also known as newly onset GD patients, were the first to be diagnosed as GD without drug therapy; refractory GD patients were treated with anti-thyroid drugs for at least 4 years and still positive for thyrotropin receptor antibody (TRAb) (17). Thyroid function test and TRAb levels of all subjects were summarized in Supplementary Table 1. This project was approved of by the Ethics Committee of Jinshan Hospital of Fudan University.

### Quantitative real-time polymerase chain reaction (qRT-PCR)

Peripheral blood monocytes (PBMCs) were separated from blood using Lymphoprep density gradient centrifugation (TianGen Biotech, China). Total RNA was extracted from PBMCs using Trizol reagent (Invitrogen, USA) according to the manufacturer's protocol. Then, the concentration of RNA was determined and 1 μg of RNA was used to synthesize cDNA by reverse transcription kit (TaKaRa, Japan). The qRT-PCR was performed in ABI PRISM 7300 Fast Real-Time PCR system (BIO-RAD) using SYBR Premix Ex TaqTM II (Perfect Real Time) (TaKaRa, Japan). The primer sequences were ATC AAT CAT CGG GTG TGG as the forward primer and AAG GCA ATA GTG ACT GGA GAC as the reversed primer for IL-36α; CAT TGC CGA CAG GAT GCA G as the forward primer and CTC GTC ATA CTC CTG CTT GCT G as the reversed primer for β-actin.

### Serum IL-36α assay

Serum samples were collected from 2 mL of EDTA-containing whole blood. After centrifuged at 4,000 rpm for 10 min, supernatants were obtained and then centrifuged at 13,000 rpm for 2 min. Serum samples were obtained and stored at −80°C. The concentration of IL-36α in serum was determined using commercial sandwich ELISA kits (CUSABIO, Wuhan, China) according to the manufacturer's instructions.

### Flow cytometry

PBMCs were stimulated with 2 μL mixture (BD Bioscience, USA) containing phorbol myristate acetate, ionomycin and monensin at 37°C and 5% CO_2_ for 4 h. Then, the cells were stained with anti-human CD4-APC at 4°C under light-free environment for 30 min. Fixation and permeabilization were conducted with a Cytofix/Cytoperm kit (BD Biosciences, USA). Then, the cells were incubated with primary antibody anti-human IL-36α (Lifespan Bioscience, USA) at 4°C for 30 min under light-free environment. The cells were sequentially washed with 1 mL BD Perm/Wash™ buffer (1 ×) (BD Bioscience, USA), and stained with secondary antibody goat anti-rabbit IgG-FITC (Santa, USA) at room temperature for 30 min. Finally, FACScalibur Flow cytometer (Beckman coulter) was used to analyze the stained cells (CD4^+^IL-36α^+^T cells) immediately.

### Cell culture

Like the previously published researches (7, 18), the separated PBMCs from GD patients or controls were divided into two equal parts (1~2 × 10^6^ /ml). Both of them were seeded into culture dishes with a diameter of 35 mm. One was incubated with recombinant human IL-2 (50 ng/ml) (PeproTech, USA) and 100 U/ml penicillin/streptomycin (Hyclone, USA) in the presence of recombinant human IL-36α (100 ng/ml) (R&D system, USA) at a 37°C incubator supplemented with 5% CO2. Another was incubated only with recombinant human IL-2 (50 ng/ml) (PeproTech, USA) and served as the negative control. After 24 h, we harvested the cultured supernatants of the both and stored them at −80°C. The levels of T-cell-derived cytokines including IFN-γ, TNF-α, IL-6 and IL-17A in supernatants were determined by Human High Sensitivity T Cell Magnetic Bead Panel (Merck Millipore, Germany) according to the manufacturer's instructions.

### Statistical analysis

All data are reported as mean ± standard deviation (M ± SD). SPSS 17.0 was used for statistical analysis. The comparison of multiple groups was conducted through the one-way ANOVA. Comparison between two groups was performed by using two-independent-sample *T* test, or nonparametric test. For the correlation of two variables, the non-parametric spearman's test was conducted. Comparison of cytokine levels in supernatant of PBMCs between stimulation with recombinant IL-36α combined with IL-2 and IL-2 only was analyzed using paired *T* test. The 2^−ΔΔ*CT*^ method was used to analyze the data of RT-PCR. Data of the levels of IFN-γ, IL-6 and TNF-α were log10 transformed. *P* < 0.05 was considered to be statistically significant difference.

## Results

### The expression of IL-36α mRNA in PBMCs

**Figure 1:**
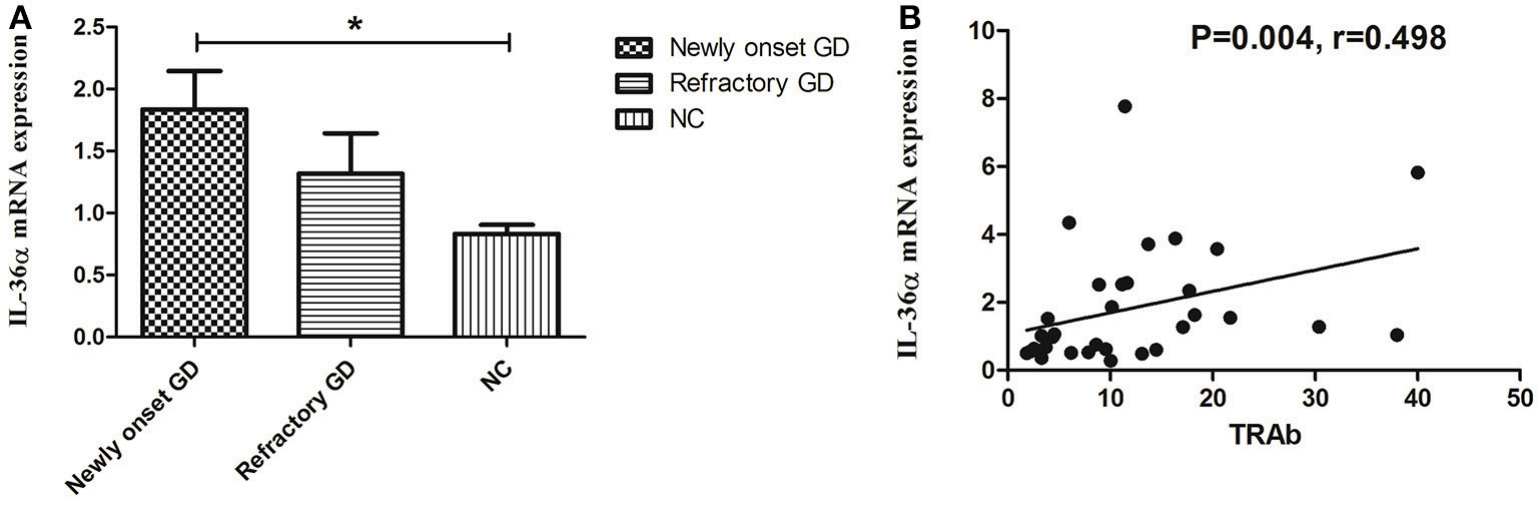
The expression of IL-36α mRNAs in GD patients **(A)**. IL-36α mRNA expression in newly onset GD patients was significantly higher than that in NC group; **(B)**. Correlation analysis of IL-36α mRNA expression and TRAb. IL-36α mRNA expression in newly onset GD patients was positively correlated TRAb. ^*^P < 0.05.

As shown in Figure 1A, the expression of IL-36α mRNA in newly onset GD patients was significant higher than that in NC group (*P* = 0.019). There was no significant difference between refractory GD and newly onset GD as well as NC (*P* > *0.05*). Correlation analysis showed that the expression of IL-36α mRNA was positively correlated with TRAb (*P* = 0.004, *r* = 0.498, Figure 1B) in newly onset GD patients. In refractory GD patients, the expression of IL-36α mRNA was not significantly correlated with free triiodothyronine (FT3), free thyroxine (FT4) and TRAb (*P* > 0.05) (data not shown).

### Serum levels of IL-36α

**Figure 2:**
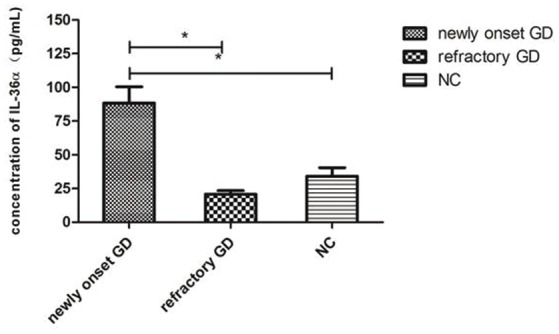
Serum IL-36α levels of GD patients (including newly onset GD, refractory patients) and healthy controls. ^*^P < 0.05.

As shown in Figure 2, the concentration of IL-36α in newly onset GD patients was significantly higher than that of refractory GD patients and NC group (*P* = 0.010; *P* = 0.007). There was no significant difference in serum IL-36α concentration between refractory GD patients and NC group (*P* = 0.406). In newly diagnosed GD group and refractory GD group, there was no significant correlation between IL-36α concentration and FT3, FT4 and TRAb (*P* > 0.05).

### Frequency of CD4^+^IL-36α^+^T cells in PBMCs

**Figure 3:**
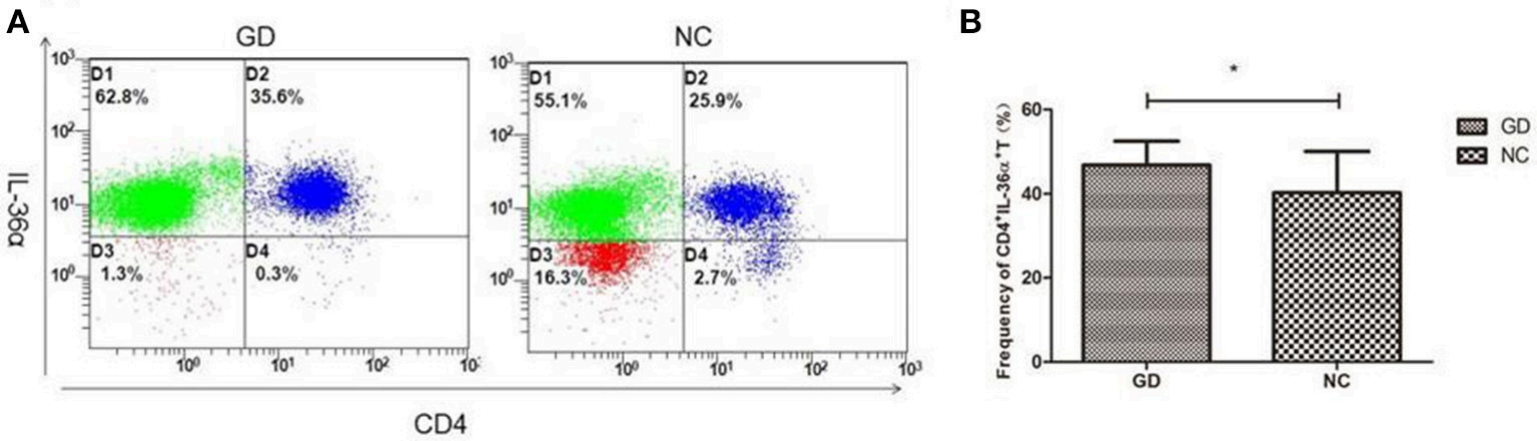
Flow cytometry analysis detected CD4^+^IL-36α^+^ T cells. **(A)** Representative flow cytometry data showing the expression of CD4^+^IL-36α^+^ T cells in newly onset GD patients and NC group. **(B)** The frequency of CD4^+^IL-36α^+^ T cells in newly onset GD patients and controls. ^*^P < 0.05.

Flow cytometric analysis revealed that the percentage of CD4^+^IL-36α^+^T cells in GD group was significantly higher than that in NC group (*P* = 0.030, Figure 3), but the percentage of CD4+IL-36α+T cells was not correlated with FT3, FT4 and TRAb (*P* > 0.05).

### The expression of cytokines in cultured PBMCs after recombinant human IL-36α stimulation

**Figure 4:**
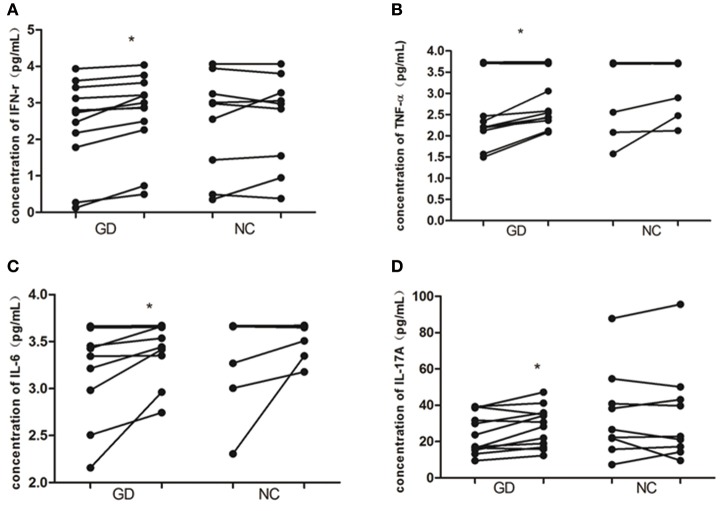
IL-36α promotes the expression of inflammatory cytokines in PBMCs of patients with GD. In GD patients, the levels of IFN-γ **(A)**, TNF-α **(B)**, IL-6 **(C)**, and IL-17A **(D)** were increased in supernatant of PBMCs stimulated with human recombinant IL-36α combined with IL-2 than that of supernatant of PBMCs stimulated with IL-2 only (*P* = 0.015, *P* = 0.016, *P* = 0.039, *P* = 0.017, respectively). For these cytokines in supernatant of PBMCs from NC, no significant difference was found between stimulation with recombinant human IL-36α combined with IL-2 and IL-2 only. Each symbol represents an individual patient or healthy control. In GD patients or NC group, the points on the left column represent the levels of cytokines after stimulation with IL-2 only; the points on the right column represent the levels of cytokines after stimulation with IL36 in combination with IL-2. ^*^P < 0.05.

As shown in Figure 4, in supernatant of PBMCs from newly onset GD patients, recombinant human IL-36α stimulation resulted in the increase of INF-γ, IL-6, IL-17A, and TNF-α (2.4 ± 1.2 pg/mL vs. 2.7 ±1.1 pg/mL, *P* = 0.015; 3.3 ± 0.5 pg/mL vs. 3.5 ± 0.3 pg/mL, *P* = 0.039; 24.2 ± 11.0 pg/mL vs. 28.2 ± 11.0 pg/mL, *P* = 0.017; 2.6 ± 0.9 pg/mL vs. 2.9 ± 0.7 pg/mL, *P* = 0.016, respectively). While the levels of INF-γ, IL-6, IL-17A, and TNF-α in PBMCs from NC group were unaffected by the stimulation of recombinant human IL-36α (*P* > 0.05).

## Discussion

The dysfunction of immune cells is a key factor in the development of GD. In recent years, the studies on immune cells and their corresponding cytokines have become a hotspot in the field of GD pathogenesis. Previous reports have demonstrated that Th1 and Th17 cells and their cytokines are abnormally expressed in autoimmune thyroid disease (19). Compared with the normal control group, the expression of Th2 cell-related cytokines such as IL-4 and IL-10 is significantly increased in GD patients (3). Similarly, protein concentrations of Th1-related cytokines such as IL-12 and IL-18 in GD patients are significantly higher than that in the normal controls (8). The percentage of Th17 cells in peripheral blood of patients with intractable GD is significantly higher than that in patients at the remission state (4). It is also confirmed that the proportion of CD4^+^CD25^+^Foxp3^+^Treg cells in the untreated GD group is significantly lower than that in the normal group and negatively correlated with the autoantibody concentration against thyroid-stimulating hormone receptor (TSHR) (5). The proportion of follicular helper T (Tfh) cells in the peripheral blood of GD patients is significantly increased, and the number of Tfh cells is positively correlated with FT3 and FT4. The proportion of circulating Tfh cells is decreased after anti-thyroid drug treatment in follow-up duration (6). Moreover, the expression of IL-22 mRNA, the concentration of serum IL-22 and the percentage of Th22 cells in PBMCs from GD patients are significantly higher than those in the normal control group (7). All those data suggest that various immune cells and cytokines may be involved in the development of GD.

IL-36α is a new member of IL-1 family (IL-1F) located on IL-1F gene cluster of human chromosome 2 (9). The IL-1 family includes seven agonists or pro-inflammatory factors (IL-1α, IL-1β, IL-18, IL-33, IL-36α, IL-36β, and IL-36r) and four inhibitors or anti-inflammatory factors (IL-1Ra, IL-36Ra, IL-37 and IL-38) (20, 21). IL-36α and IL-IRa have 24% homology (22). IL-36α can induce the production of pro-inflammatory cytokines, chemokines and costimulatory molecules, and then accomplish the recruitment of neutrophils, the activation of dendritic cells and the polarization of Th1 cells (16, 23).

The abnormal expression of IL-36α has been found in a series of autoimmune inflammatory diseases such as psoriasis, rheumatoid arthritis, and Crohn's disease (24). IL-36RN is a gene encoding the native inhibitor for IL-36, and is deficient in severe pustular psoriasis (25). IL-36α can up-regulate the expression of IL-17A, IL-23, and tumor necrosis factor (TNF-α) in psoriasis arthritis (PsA) (26). The expression of IL-36α in the lesion skin is significantly higher than that in the normal skin of psoriasis (27). IL-36 can also induce the secretion of IL-6 and IL-8 in the culture of synovial cells (28). The increased level of IL-36α in serum from patients with rheumatoid arthritis is positively correlated with C-reactive protein (CRP) (29). The elevated expression of IL-36α in patients with Sjogren's syndrome is positively associated with the expression of IL-22, IL-17 and IL-23p19 in the lip gland tissues (30). The higher level of IL-36α in serum of patients with systemic lupus erythematosus (SLE) is also positively correlated with disease procession (31). Upon the stimulation with recombinant IL-36α and IL-36γ, the concentrations of IL-6 and CXCL8 in the culture of PBMCs are significantly increased when compared with NC (31).

In the present study, the expression of IL-36α in PBMCs and serum of newly diagnosed GD patients was significantly higher than that of NC group. This is consistent with the results of previous studies on the association of IL-36 with Sjogren's syndrome (30) and SLE (31). Correlation analysis showed that the expression of IL-36α mRNA in newly diagnosed GD patients was positively correlated with TRAb, suggesting that IL-36α was involved in the development of GD and correlated with the severity of the disease. There was no significant difference in IL-36α mRNA in PBMCs and level of IL-36α in serum between refractory GD patients and NC group. The reason may be that refractory GD patients take drugs for a long time, thereby the expression of IL-36α was inhibited to some extent. Flow cytometric assay showed that the percentage of CD4^+^IL-36α^+^ T cells in PBMCs from newly onset GD patients was higher than that in normal controls, implicating that CD4^+^ T cells are one of the major cells for the production of IL-36α in GD. The up-regulation of IL-36α in serum from newly diagnosed GD patients may be associated with an increase in the percentage of CD4^+^T cells secreting IL-36α in PBMCs.

The PBMCs stimulation test showed that the expression of IFN-γ, TNF-α, IL-6, and IL-17A in the supernatant of PBMCs from GD patients was significantly increased after stimulation with human recombinant IL-36α, while the concentrations of above cytokines in the culture supernatant did not reveal a significant change in NC group. These results suggest that IL-36α may participate in the process of immune disorder in GD by acting on Th1, Th2, and Th17 cells and inducing the secretion of inflammatory cytokines. However, our results were not consistent with the results from a previous study about IL-36α in SLE with the only elevated level of IL-6, instead of IFN-γ and IL-17A (31), which may be related to IL-36α concentration, detection kit and cell population because the target cells and the intensity of IL-36α are different in different autoimmune inflammatory diseases.

In summary, the pro-inflammatory factor IL-36α is involved in GD pathogenesis, which is beneficial to elucidate the pathogenic mechanisms of GD and to develop a new immune-specific therapy.

## Ethics statement

This study was carried out in accordance with the recommendations of human participants, Ethics Committee of the Jinshan Hospital of Fudan University with written informed consent from all subjects. All subjects gave written informed consent in accordance with the Declaration of Helsinki. The protocol was approved by the Ethics Committee of the Jinshan Hospital of Fudan University committee.

## Author contributions

QY carried out the work, conducted the data analysis and wrote the manuscript. LL, Z-YS, BW, QQ, XA helped with the collection of specimens. JZ revised the manuscript.

### Conflict of interest statement

The authors declare that the research was conducted in the absence of any commercial or financial relationships that could be construed as a potential conflict of interest.
