# Supplementary material for: Elevated Interleukin-36α And CD4+IL-36α+T Cells Are Involved in the Pathogenesis of Graves' Disease
Source: Front Endocrinol (Lausanne). 2018 Oct 9;9:591. doi: 10.3389/fendo.2018.00591 (PMC6190875; doi:10.3389/fendo.2018.00591)
Supplement: Supplementary file 1 [file Table_1.docx]

**Supplementary Table 1 Clinical data of all subjects**

| Method | Group | N  (M/F) | Age  (years) | FT3  (pmol/L) | FT4  (pmol/L) | TSH  (mIU/L) | TRAb  (IU/L) |
| --- | --- | --- | --- | --- | --- | --- | --- |
| qRT-PCR | Newly onset GD | 32 (7/25) | 37.6±13.7 | 24.3±13.2 | 51.0±27.4 | <0.005 | 12.3±9.7 |
|  | Refractory GD | 15 (3/12) | 33.6±11.1 | 8.2±7.9 | 21.5±17.6 | 0.6±0.8 | 9.3±9.1 |
|  | Controls | 30 (8/22) | 35.2±12.3 | - | - | - | - |
| ELISA | Newly onset GD | 46 (15/31) | 43.8±12.4 | 22.6±12.2 | 54.7±34.5 | <0.005 | 13.3±11.9 |
|  | Refractory GD | 10 (5/5) | 41.3±9.4 | 12.2±10.5 | 26.7±18.0 | 0.3±0.05 | 12.3±10.0 |
|  | Controls | 24(8/16) | 40.7±9.8 | - | - | - | - |
| Flow cytometry | Newly onset GD | 19 (3/16) | 41.0±9.2 | 18.5±11.9 | 51.1±28.5 | <0.005 | 9.9±11.6 |
|  | Controls | 10 (2/8) | 38.2±6.0 | - | - | - | - |
| Cell cuture | Newly onset GD | 12 (4/8) | 36.7±11.6 | 15.0±18.0 | 35.2±34.6 | <0.005 | 16.6±12.7 |
|  | Controls | 9 (2/7) | 35.3±9.0 | - | - | - | - |

(Data were expressed as M±SD; M, Male; F, Female; FT3, free Triiodothyronine; FT4, free Thyroxine; TSH, thyroid-stimulating hormone; TRAb, thyrotropin receptor antibody; qRT-PCR, quantitative real-time polymerase chain reaction; ELISA, enzyme linked immunosorbent assay)
